# Supplementary material for: Structural basis for amino acid transport by the CAT family of SLC7 transporters
Source: Nat Commun. 2018 Feb 7;9:550. doi: 10.1038/s41467-018-03066-6 (PMC5803215; doi:10.1038/s41467-018-03066-6)
Supplement: Supplementary file 1 — Supplementary Information [file 41467_2018_3066_MOESM1_ESM.pdf]

Structural basis for amino acid transport by the CAT family of SLC7 transporters.

Jungnickel, *et al*.

Supplementary Figure 1

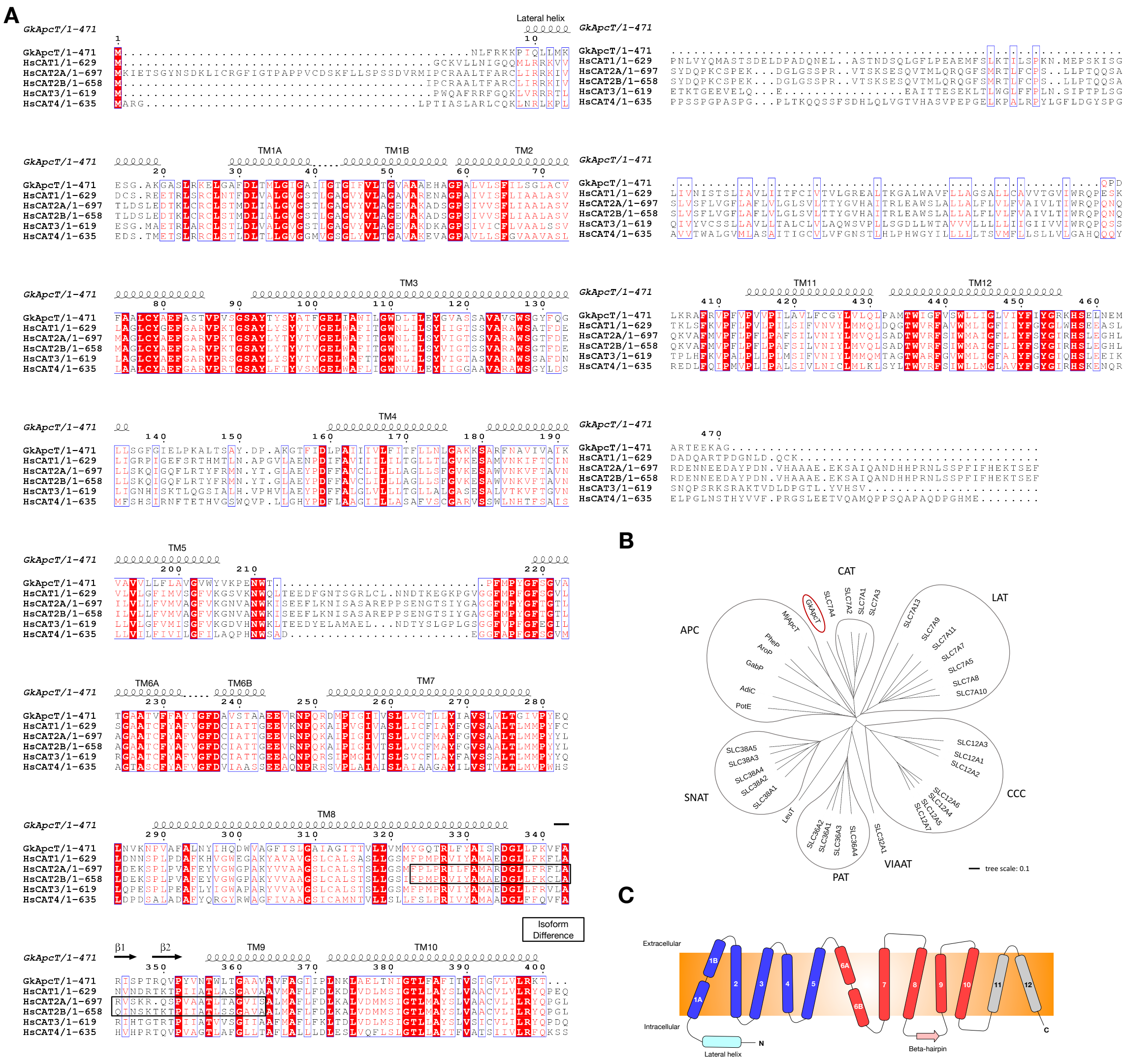

Supplementary Figure 1. Multiple sequence alignment of GkApcT with the human cationic amino acid transporters. A. GkApcT is shown aligned against human CAT-1 (SLC7A1; Uniprot P30825), CAT-2A (SLC7A2; Uniprot P52569-2), CAT-2B (SLC7A2; Uniprot P52569-1), CAT-3 (SLC7A3; Uniprot Q8Wy08) and CAT-4 (SLC7A4; Uniprot Q43246). The transmembrane helices are shown above the alignment for GkApcT. **B.** Phylogenetic tree showing the relationship between GkApcT and other APC family transporters. **C.** Topology diagram of GkApcT. The 5+5 inverted topology structure is shown in blue and red respectively, with TM11 and 12 shown in grey. The lateral helix and beta hairpin are also indicated.

Supplementary Figure 2

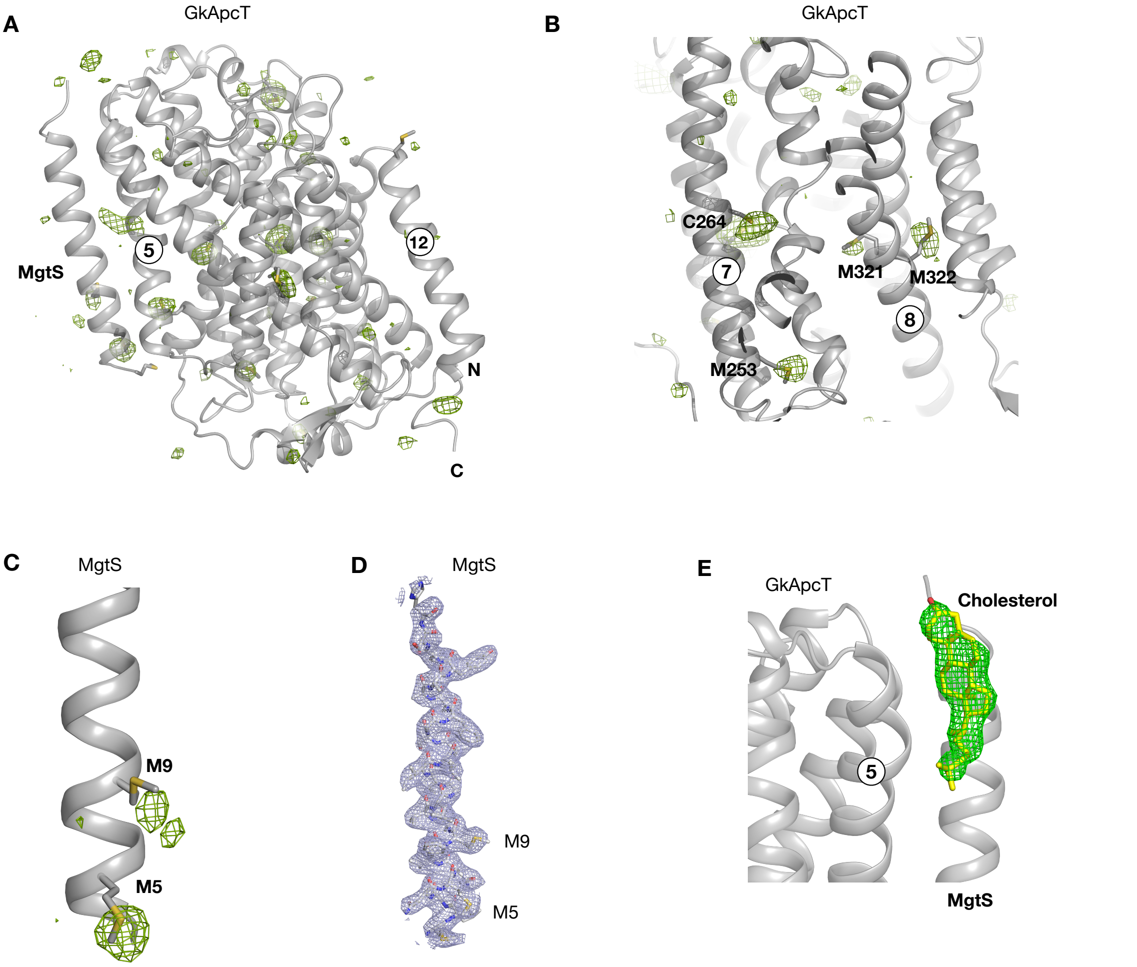

Supplementary Figure 2. Model building and structure validation using long wavelength sulphur SAD data. **A.** Crystal structure of GkApcT with the anomalous difference density peaks for sulphur atoms, calculated from the long wavelength data collected at 2.7 Å wavelength (Table 1). **B.** Zoomed in view of the structure, showing the peaks for several methionine and cysteine residues, including Met321. **C.** Anomalous difference peaks for sulphur atoms on YneM, contoured at 3σ. **D.** 2mFo-DFc electron density, contoured at 1σ, after refinement of the YneM helix. **E.** Difference electron density (mFo-DFc, contoured at 2σ) observed in the cholesterol binding pocket formed between GkApcT and YneM in the crystal structure.

Supplementary Figure 3

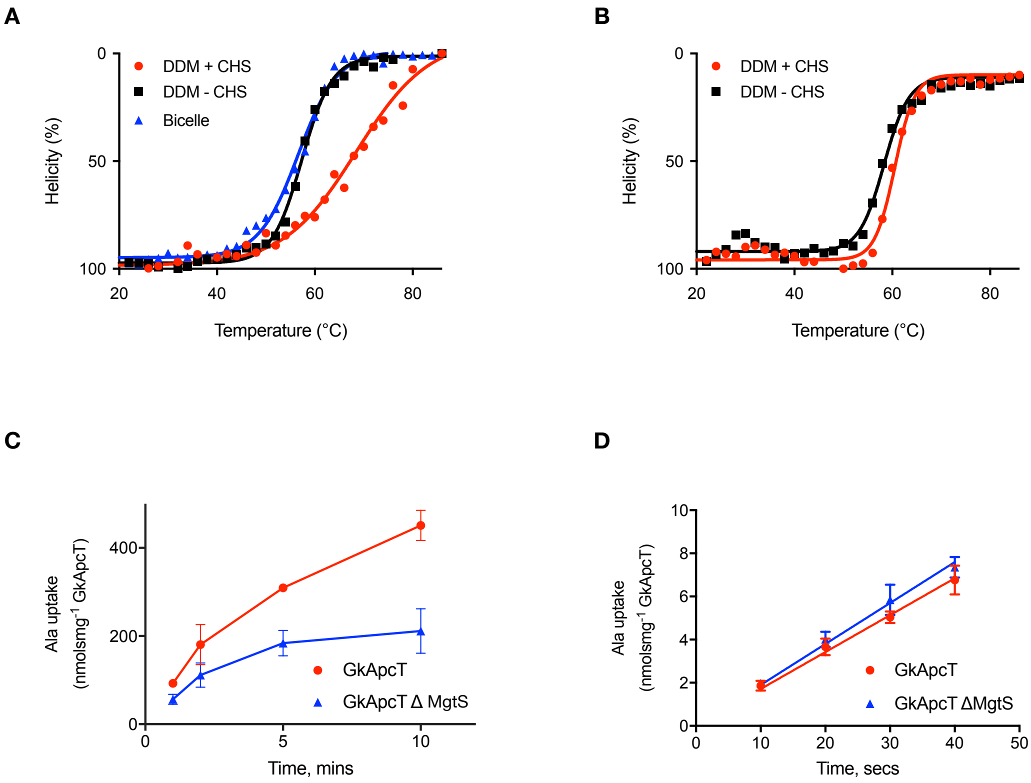

Supplementary Figure 3. Analysis of thermal stability and transport properties of GkApcT purified from C43 (DE3) and the Δ YneM KO strain. A. Thermal stability assays showing the effect of cholesterol hemisuccinate on the stability of GkApcT-YneM. **B.** Stability of GkApcT purified from the ΔYneM KO strain of *E. coli*. **C.** Time dependent uptake of 3H L-Ala by GkApcT purified from either C43 (DE3) (red) or the Δ YneM KO strain of C43 (DE3) (blue). **D.** Initial rates of accumulation for samples shown in C. n=3 independent experiments, error bars s.d.

Supplementary Figure 4

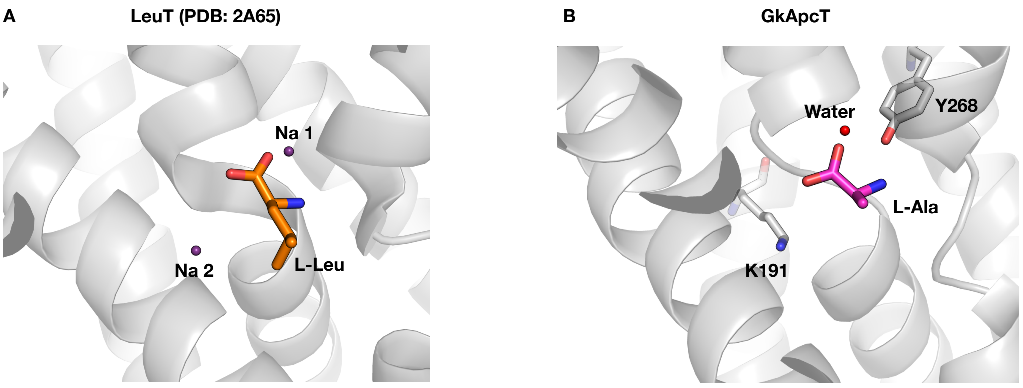

Supplementary Figure 4. Comparison of the amino acid binding site between LeuT and GkApcT. A. View of the binding site it LeuT (PDB 2A65), with the bound L-leucine shown in sticks and sodium ions as purple spheres. **B.** Equivalent view of the binding site in GkApcT.

Supplementary Figure 5

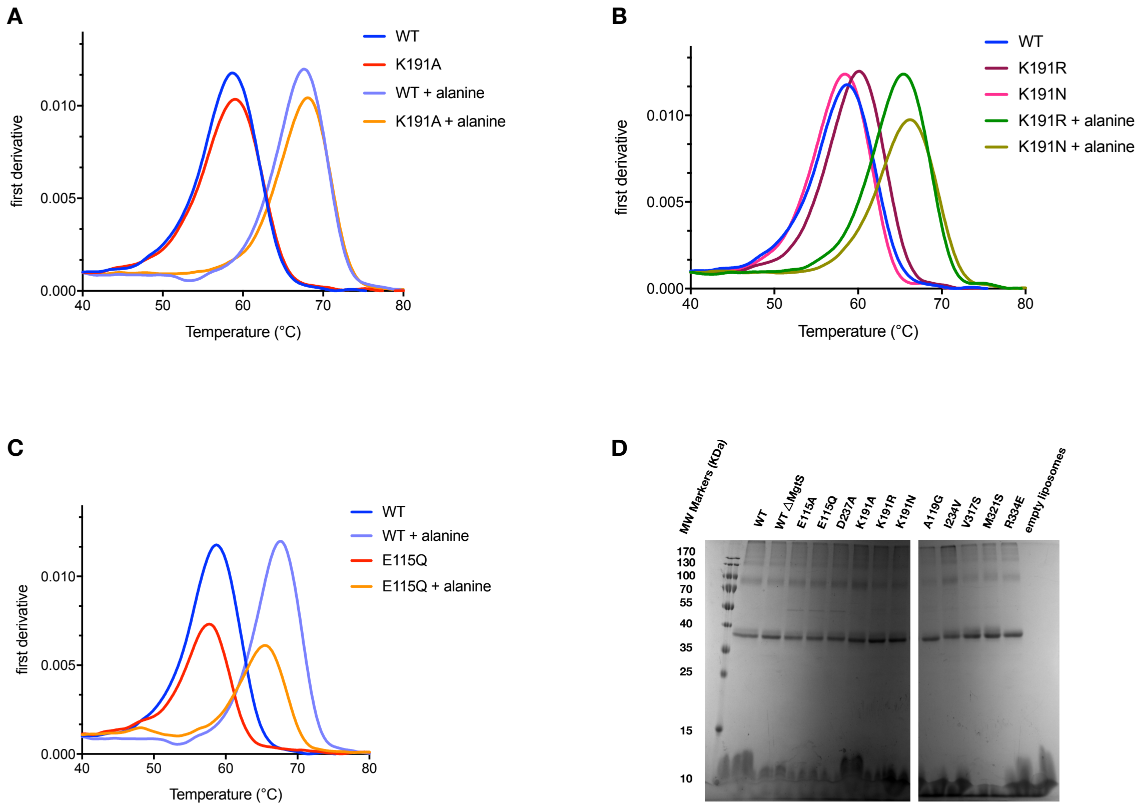

Supplementary Figure 5. Analysis of thermal stability in the presence of ligand for GkApcT WT and mutant variants. A. Thermal stability assays using a Prometheus NT.48 showing the stabilising effect of the presence of 10 mM alanine on both wild type GkApcT and the Lys191Ala variant. **B.** Increased stability of the Lys191 arginine and asparagine variants in the presence of 10 mM alanine. **C.** Analysis of Glu115Gln variant. **D.** SDS-PAGE (12% Tris-glycine) analysis of WT and reconstituted variants of GkApcT used in the study.

Supplementary Figure 6

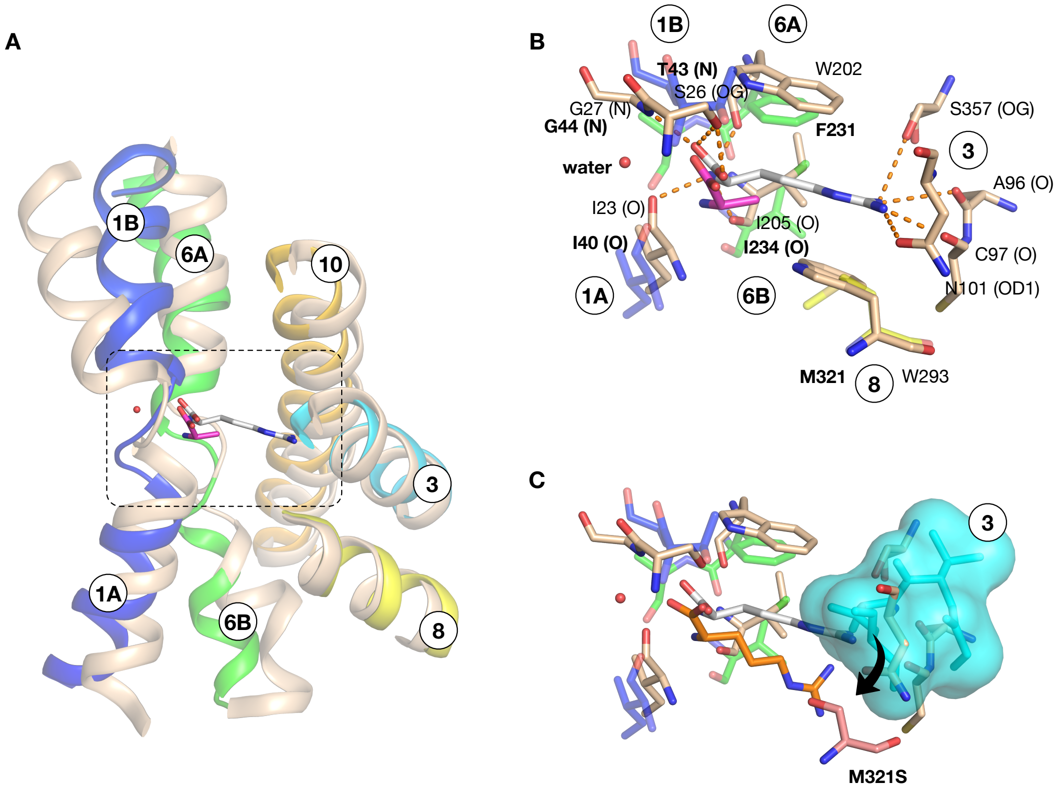

Supplementary Figure 6. Structural comparison between GkApcT and L-Arginine bound AdiC structure (PDB 3L1L). A. Overlay of AdiC (wheat) on the L-Alanine bound GkApcT structure (coloured as in Figure 1). Helices are indication and the bound L-Arginine in AdiC shown as grey sticks. **B.** Close up view of the area highlighted in the dashed box in A. Residue numbers in GkApcT are in bold, the equivalent residues in AdiC are not in bold. The residues contributed by AdiC are shown in wheat, those from GkApcT coloured as in A. Residue numbers from GkApcT are in bold, those from AdiC in normal text. **C.** Equivalent view as B, with the residues from TM 3 in GkApcT shown as space fill in cyan. TM 3 encroaches into the binding site forcing the guanidinium group of L-Arg down relative to its position in AdiC (movement shown using the black arrow). A potential interaction between the guanidinium group in the mammalian CATs could be made to the conserved serine on TM8.

Supplementary Table 1

	**Sequence 5'-3'**
pWaldo_GFPd_Forward	GGAATTCCATATGAATTTGTTTCGTAAAAAAC
pWaldo_GFPd_Reverse	CGCGGATCCTCCAGCCTTTTCTTCCGTCCG
E115A_F	GGGATTTGATTTTGGCGTACGGGGTTGCTTCGTCGG
E115A_R	CCGACGAAGCAACCCCGTACGCCAAAATCAAATCCC
E115Q_F	GGGATTTGATTTTGCAGTACGGGGTTGCTTCGTCGG
E115Q_R	CCGACGAAGCAACCCCGTACTGCAAAATCAAATCCC
A119G_F	CGGGGTTGCTTCGTCGGGCGTCGCTGTTGGTTGGTCCG
A119G_R	CGGACCAACCAACAGCGACGCCCGACGAAGCAACCCCG
K191A_F	GCGGTGATCGTTGCCATTGCCGTGGCGGTTGTGCTG
K191A_R	CAGCACAACCGCCACGGCAATGGCAACGATCACCGC
K191N_F	GCGGTGATCGTTGCCATTAACGTGGCGGTTGTGCTG
K191N_R	CAGCACAACCGCCACGTTAATGGCAACGATCACCGC
K191R_F	GCGGTGATCGTTGCCATTCGAGTGGCGGTTGTGCTG
K191R_R	CAGCACAACCGCCACTCGAATGGCAACGATCACCGC
I234V_F	CGGTGTTTTTCGCTTACGTGGGCTTTGATGCGGTGTCG
I234V_R	CGACACCGCATCAAAGCCCACGTAAGCGAAAAACACCG
D237A_F	GCTTACATCGGCTTTGCGGCGGTGTCGACGGCGG
D237A_R	CCGCCGTCGACACCGCCGCAAAGCCGATGTAAGC
V317S_F	CGCCGGCATTACGACCAGCCTCCTTGTGATGATGTATGG
V317S_R	CCATACATCATCACAAGGAGGCTGGTCGTAATGCCGGCG
M321S_F	CGACCGTGCTCCTTGTGAGCATGTATGGGCAGACGCG
M321S_R	CGCGTCTGCCCATACATGCTCACAAGGAGCACGGTCG

Supplementary Table 1. Sequences of the primers used in this study.
